# A non-lab nomogram of survival prediction in home hospice care patients with gastrointestinal cancer

**DOI:** 10.1186/s12904-020-00690-2

**Published:** 2020-12-07

**Authors:** Muqing Wang, Xubin Jing, Weihua Cao, Yicheng Zeng, Chaofen Wu, Weilong Zeng, Wenxia Chen, Xi Hu, Yanna Zhou, Xianbin Cai

**Affiliations:** 1 Department of Gastroenterology, The First Affiliated Hospital of Shantou University Medical College, 57 Changping Road, Shantou, Guangdong 515041 People’s Republic of China; 2 Department of Hospice, The First Affiliated Hospital of Shantou University Medical College, Shantou, Guangdong 515041 People’s Republic of China

**Keywords:** Home-based hospice, Gastrointestinal cancer, Nomogram, LASSO, Prognosis of survival

## Abstract

**Background:**

Patients suffering from gastrointestinal cancer comprise a large group receiving home hospice care in China, however, little is known about the prediction of their survival time. This study aimed to develop a gastrointestinal cancer-specific non-lab nomogram predicting survival time in home-based hospice.

**Methods:**

We retrospectively studied the patients with gastrointestinal cancer from a home-based hospice between 2008 and 2018. General baseline characteristics, disease-related characteristics, and related assessment scale scores were collected from the case records. The data were randomly split into a training set (75%) for developing a predictive nomogram and a testing set (25%) for validation. A non-lab nomogram predicting the 30-day and 60-day survival probability was created using the least absolute shrinkage and selection operator (LASSO) Cox regression. We evaluated the performance of our predictive model by means of the area under receiver operating characteristic curve (AUC) and calibration curve.

**Results:**

A total of 1618 patients were included and divided into two sets: 1214 patients (110 censored) as training dataset and 404 patients (33 censored) as testing dataset. The median survival time for overall included patients was 35 days (IQR, 17–66). The 5 most significant prognostic variables were identified to construct the nomogram among all 28 initial variables, including Karnofsky Performance Status (KPS), abdominal distention, edema, quality of life (QOL), and duration of pain. In training dataset validation, the AUC at 30 days and 60 days were 0.723 (95% CI, 0.694–0.753) and 0.733 (95% CI, 0.702–0.763), respectively. Similarly, the AUC value was 0.724 (0.673–0.774) at 30 days and 0.725 (0.672–0.778) at 60 days in the testing dataset validation. Further, the calibration curves revealed good agreement between the nomogram predictions and actual observations in both the training and testing dataset.

**Conclusion:**

This non-lab nomogram may be a useful clinical tool. It needs prospective multicenter validation as well as testing with Chinese clinicians in charge of hospice patients with gastrointestinal cancer to assess acceptability and usability.

## Background

Cancer is currently considered as an important cause of mortality around the globe. According to the latest estimated result of global cancer burden, the incidence and mortality in China account for 23.7 and 30.2% of cancer in the world respectively [1]. Moreover, the incidence and mortality cases of gastrointestinal cancer such as esophageal cancer, gastric cancer, and liver cancer in China make up about half of that observed globally [2]. Most patients with gastrointestinal cancer are at the advanced stage when diagnosed [3]. For treatment-refractory disease and as functional decline begins, patients can benefit from hospice care for symptom management and to reduce suffering at the end of life. When someone is choosing hospice, predicting survival time is more important than predicting treatment response, as it provides opportunity for patients and families to achieve closure [4, 5]. The dying trajectory of patients with cancers is part of the most predictable prognostic information [6]. However, previous studies consistently reported about the inaccuracy of clinicians in estimating the survival time and mainly rely on their intuitions or self-clinical judgment [7, 8]. Systematic reviews have shown that clinicians often overestimated actual life expectancy [9, 10].

To improve the accuracy of clinicians’ predictions, numerous prediction tools have been designed specifically for advanced-stage patients. These tools were the Palliative Prognostic (PaP) score [11], Delirium-PaP (D-PaP) [12], Palliative Performance Scale (PPS) [13, 14], Palliative Prognostic Index (PPI) [15], modified Glasgow Prognostic Score (mGPS) [16, 17], and Prognosis in Palliative Care Study (PiPS) [18] etc. However, there is no consensus regarding the most appropriate tool for clinical use [19]. Therefore, many studies have further determined prognostic factors in terminal cancer patients and constructed specific survival prediction models. For example, Feliu et al. produced an exceedingly accurate nomogram that uses basic clinical and analytical information to predict the probability of survival at 15, 30, and 60 days in terminally ill cancer patients [20]. Schonwetter et al. performed statistical analysis on data from more than 300 terminal lung cancer patients in a non-profit community hospice to develop a lung cancer-specific prognostic tool to predict 50 and 90% mortality in the days after admission to a hospice [21]. As far as we know, there is no gastrointestinal cancer-specific prognostic model for home hospice patients in China.

Compared with developed countries, China’s hospice career started late and developed more slowly. In China, as an important developing country, only a few investigators and institutions participate in hospice care-related research, especially for prognostic survival. As a result, China has lost the opportunity to share and exchange experiences with the world in the field of hospice [22]. Wang YM et al. performed a follow-up study on 674 patients with advanced stages of cancer in a hospice and identified factors that significantly affect the survival rate [23]. Zhou LJ et al. retrospectively analyzed data from 1019 advanced cancer patients who died within six months in a palliative home care service and produced a simple Chinese Prognostic Scale (ChPS) for predicting the survival rate of patients with an advanced stage of cancer [24]. In summary, there is a scarcity of studies concerning the survival of gastrointestinal cancer patients receiving home hospice care service in China as well as its predictors. Moreover, the least absolute shrinkage and selection operator (LASSO) Cox regression, with advantages of building predictive models that are more accurate, robust, and generalizable [25], has not been used in these patients. Thus, the aim of our study was to utilize LASSO Cox regression to build a model to accurately predict survival time in home hospice care patients with gastrointestinal cancer. In addition, we constructed a nomogram to represent our predictive model in a graphical format, making the model more accessible to clinicians and patients alike.

## Methods

### Research objects

Patients with gastrointestinal cancer who survived less than six months from the Hospice Unit of Shantou University Medical College-affiliated First Hospital between January 2008 and December 2018 were included in this retrospective study. The Hospice Unit of Shantou University Medical College-affiliated First Hospital is the first Hospice Unit established in 1998, founded by Li Ka Shing Foundation to provide free home-based holistic care for patients with terminal cancer in mainland China [26]. Patients with any missing data were excluded from our study. The current study includes the retrospective statistical analysis on clinical data of the deceased patients, without disclosing the patients’ identity, and signed consent was not obtained, in accordance with the guidelines of the Chinese Ministry of Health.

### Data collection

The following data were collected from the case records: (1) general baseline characteristics—including age, sex, area of residence (rural or urban), education, survival time, awareness of the disease (full understanding/partial understanding/ complete ignorance), hypertension history, diabetes history, smoking history, drinking history; (2) disease-related characteristics—including cancer diagnosis, metastasis, previous cancer treatment (surgery/chemotherapy/radiotherapy), duration of pain before admission, related major symptom (constipation/anorexia/nausea/vomiting/abdominal distention/weight loss/insomnia/edema/tachypnea), previous analgesic treatment (none/NSAIDs/weak opioids/strong opioids), and effect of previous analgesic treatment (none/bad/average/satisfied); (3) related assessment scale score—including Karnofsky Performance Scale (KPS) score, quality-of-life (QOL) score, and numeric rating scale (NRS) score. These data were recorded by the clinical team, consisting of four physicians and two nurses, on a series of structured data collection sheets on admission. The survival time was calculated as the number of days from admission to an event (dead or service paused). The symptoms were collected as “present” or “absent” on admission. The degree of pain was assessed by numeric rating scale (NRS) [27]: 0 for painless, 1–3 for mild pain, 4–6 for moderate pain, and 7–10 for severe pain. The patient’s performance status was evaluated according to the Karnofsky Performance Scale (KPS) [28, 29], an 11-point rating scale that ranges from normal functioning (100) to dead (0), which has been translated into Chinese. The QOL scale (Chinese version), consisting of 12 items—including appetite, energy, attitude toward treatment, sleep, family relationships, fatigue, work relationships, pain, perception of cancer, activities of daily life, side effects of treatment and facial expression, was developed by Sun Yan in the 1990s by applying international scales to the context of the Chinese culture [24]. The total score for this scale is 60, with 1–5 scores for each item. For instance, the appetite is scored from hardly eat (1) to normally eat (5).

### Statistical analysis

The data were split into two sets using stratified random sampling: 75% as a training set for developing a predictive model and 25% as a testing set for validating it. The differences between the testing and training sets were evaluated using the Mann-Whitney U test for continuous variables and the chi-square test for categorical variables. Categorical variables were represented as percentages while continuous variables were reported as median and interquartile ranges (IQR). Before performing statistical analyses, we converted variables including KPS scores, QOL scores, NRS scores and age into categorical variables by using the X-tile software (version 3.6.1, http://medicine.yale.edu). X-tile plots provide an intuitive method to assess the association between variables and survival. The X-tile program can automatically select the optimum data cut point according to the highest χ^2^ value (minimum *p* value) defined by Kaplan–Meier survival analysis and log-rank test [30]. As a result, we categorized KPS scores as 30 or lesser, 40, 50 or more; QOL scores as 30 or lesser, 31or more; NRS scores as 3 or lesser, 4 to 6, 7 or more; age as less than 60 years, 60 or more years.

We used the 10-fold cross-validated Cox proportional hazard regression with LASSO-penalization to select the most significant prognostic variables of all initial variables. By performing both variable selection and penalization, the LASSO is able to build accurate models without under-fitting or overfitting, which leads to superior performance over traditional multivariable regression [31]. Consequently, the LASSO has been extended and broadly applied to the Cox proportional hazard regression model for survival analysis [32]. Further, the most significant predictors were identified to construct the nomogram to predict the 30-day and 60-day survival probability by using multivariate Cox regression. In other words, we used the Cox regression model to do the multivariable survival analysis, and Cox regression coefficients to generate the nomogram [33]. For multivariate analysis of survival probability, the Cox regression was performed with the forward stepwise procedure. Then, the performance of the nomogram was evaluated using the area under receiver operating characteristic curve (AUC) along with a 95% confidence interval and calibration curves (500 bootstrap resamples) in both the training and testing dataset. The AUC value is almost treated as C-statistics to evaluate the predicting performances dynamically and more intuitively [34, 35]. And calibration curve is useful for assessing whether predicted outcomes approximate actual outcomes.

The R software version 3.6.2 (https://www.r-project.org/) was used for the statistical analysis and *P* < 0.05 was considered as the statistically significant. The overall survival analysis was performed by Kaplan-Meier using “survival” and “survminer” packages. LASSO Cox regression analysis and nomogram were operated with the “glmnet” and “rms” packages. Receiver operating characteristic curves and calibration curves analysis was conducted using the “timeROC” and “rms” packages. A table for baseline patient characteristics was generated using the “tableone” package.

## Results

### Characteristics of the dataset

**Fig. 1:**
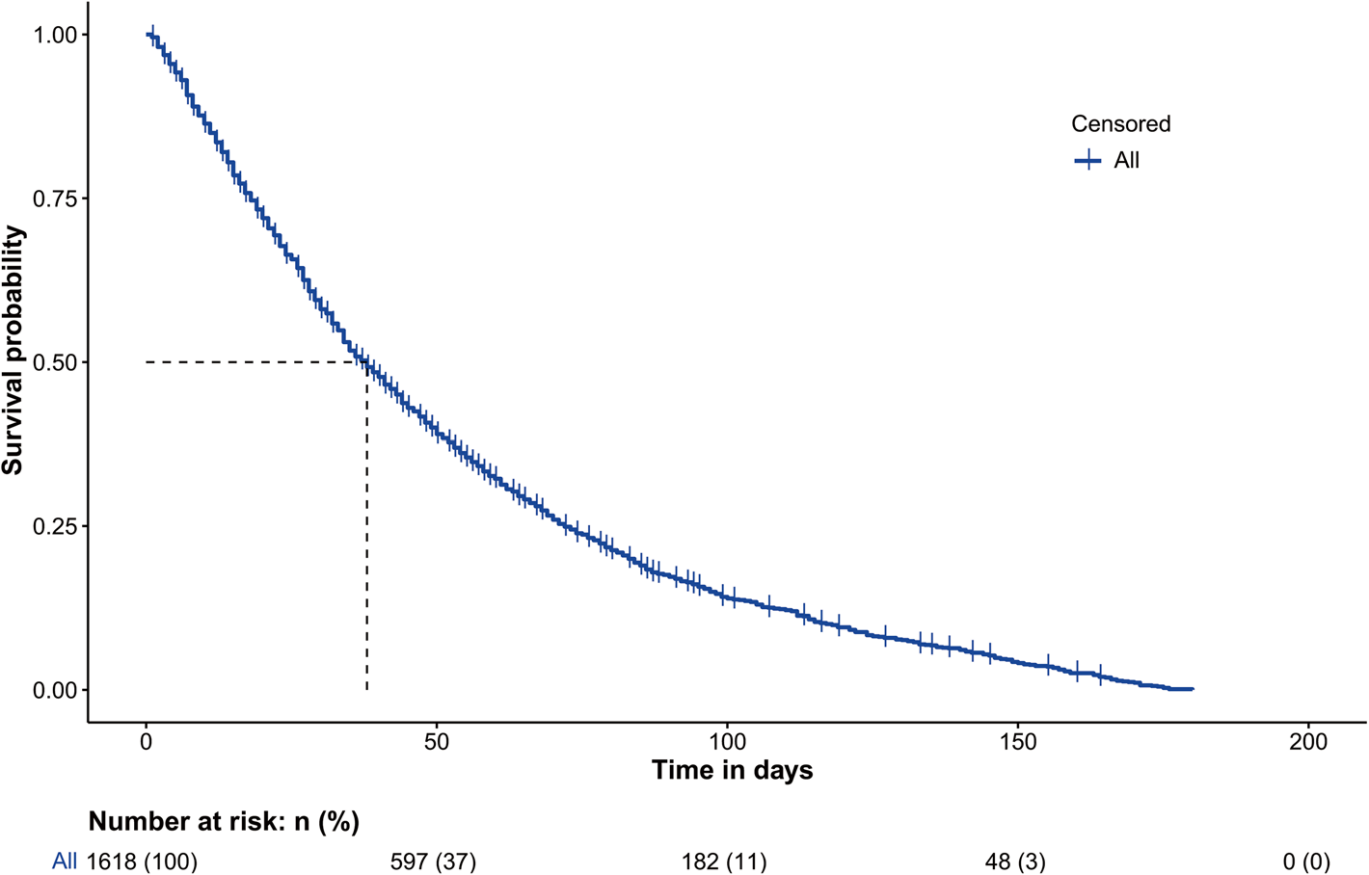
The survival curve for patients with gastrointestinal cancer in home palliative care

181 patients with any missing data were excluded from our study analysis. After exclusion, a total of 1618 patients with gastrointestinal cancer were included in our study and randomly divided into two sets: 1214 patients (110 censored) as training dataset and 404 patients (33 censored) as testing dataset. The overall survival function with a risk table was shown in Fig. 1. The median survival time for overall included patients was 35 days (interquartile ranges [IQR], 17–66). Among all cases, 70.3% were men and 57.8% were older than 60 years. Detailed information between the training and testing dataset were summarized in Table 1. The median survival time for training dataset and testing dataset was 35 days (interquartile ranges [IQR], 17–66) and 34 days (interquartile ranges [IQR], 18–68), respectively. As shown in Supplementary Table 1, Liver cancer (25.3%), esophageal cancer (24.8%), colorectal cancer (23.8%) and gastric cancer (14.8%) accounted for the vast majority of all included cases. On the whole, there was no much difference between the training and testing dataset.

**Table 1:** Patient characteristics between the training and testing dataset

Patient characteristic	Training set	Testing set	P	Patient characteristic	Training set	Testing set	P
No. of patients	1214	404		Surgery (%)			0.792
Survival time (median [IQR]), days	35.00 [17.00, 66.00]	34.00 [18.00, 68.00]	0.844	N	680 (56.0)	230 (56.9)	
Sex (%)			0.037	Y	534 (44.0)	174 (43.1)	
Female	378 (31.1)	103 (25.5)		Chemotherapy (%)			0.946
Male	836 (68.9)	301 (74.5)		N	777 (64.0)	260 (64.4)	
Age (%)			0.103	Y	437 (36.0)	144 (35.6)	
< 60 years	527 (43.4)	156 (38.6)		Radiotherapy (%)			0.038
≥ 60 years	687 (56.6)	248 (61.4)		N	1085 (89.4)	345 (85.4)	
Area of residence (%)			0.083	Y	129 (10.6)	59 (14.6)	
Rural	379 (31.2)	107 (26.5)		Duration of pain (%)			0.247
Urban	835 (68.8)	297 (73.5)		< 1 month	259 (21.3)	102 (25.2)	
Education (%)			0.863	1–6 months	831 (68.5)	260 (64.4)	
Illiteracy	163 (13.4)	61 (15.1)		6–12 months	99 (8.2)	30 (7.4)	
Primary school	589 (48.5)	199 (49.3)		> 12 months	25 (2.1)	12 (3.0)	
Middle school	292 (24.1)	89 (22.0)		Previous analgesic treatment (%)			0.642
High school	133 (11.0)	42 (10.4)		None	349 (28.7)	116 (28.7)	
High school above	37 (3.0)	13 (3.2)		NSAIDs	123 (10.1)	50 (12.4)	
Awareness of the disease (%)			0.121	Weak Opioids	373 (30.7)	119 (29.5)	
Full understanding	675 (55.6)	212 (52.5)		Strong Opioids	369 (30.4)	119 (29.5)	
Partial understanding	191 (15.7)	55 (13.6)		Effect (%)			0.995
Complete ignorance	348 (28.7)	137 (33.9)		None	280 (23.1)	94 (23.3)	
Metastasis (%)			0.468	Bad	163 (13.4)	54 (13.4)	
N	260 (21.4)	79 (19.6)		Average	618 (50.9)	207 (51.2)	
Y	954 (78.6)	325 (80.4)		Satisfied	153 (12.6)	49 (12.1)	
Hypertension (%)			0.517	Vomiting (%)			0.02
N	1031 (84.9)	337 (83.4)		N	825 (68.0)	300 (74.3)	
Y	183 (15.1)	67 (16.6)		Y	389 (32.0)	104 (25.7)	
Diabetes (%)			0.231	Abdominal distention (%)			0.684
N	1108 (91.3)	360 (89.1)		N	808 (66.6)	274 (67.8)	
Y	106 (8.7)	44 (10.9)		Y	406 (33.4)	130 (32.2)	
Smoke (%)			0.978	Tachypnea (%)			0.352
N	1025 (84.4)	342 (84.7)		N	937 (77.2)	302 (74.8)	
Y	189 (15.6)	62 (15.3)		Y	277 (22.8)	102 (25.2)	
Drink (%)			0.83	Edema (%)			0.599
N	1112 (91.6)	368 (91.1)		N	969 (79.8)	328 (81.2)	
Y	102 (8.4)	36 (8.9)		Y	245 (20.2)	76 (18.8)	
Constipation (%)			0.731	NRS (%)			0.152
N	690 (56.8)	225 (55.7)		0–3	126 (10.4)	55 (13.6)	
Y	524 (43.2)	179 (44.3)		4–6	664 (54.7)	205 (50.7)	
Weight loss (%)			0.201	7–10	424 (34.9)	144 (35.6)	
N	101 (8.3)	25 (6.2)		KPS (%)			0.313
Y	1113 (91.7)	379 (93.8)		≤30	375 (30.9)	141 (34.9)	
Insomnia (%)			0.062	40	531 (43.7)	169 (41.8)	
N	597 (49.2)	221 (54.7)		≥50	308 (25.4)	94 (23.3)	
Y	617 (50.8)	183 (45.3)		QOL (%)			0.416
Anorexia (%)			0.684	≤30	450 (37.1)	140 (34.7)	
N	168 (13.8)	52 (12.9)		> 30	764 (62.9)	264 (65.3)	
Y	1046 (86.2)	352 (87.1)		Status (%)			0.655
Nausea (%)			0.328	Censored	110 (9.1)	33 (8.2)	
N	835 (68.8)	289 (71.5)		Dead	1104 (90.9)	371 (91.8)	
Y	379 (31.2)	115 (28.5)					

### Nomogram construction and validation

**Fig. 2:**
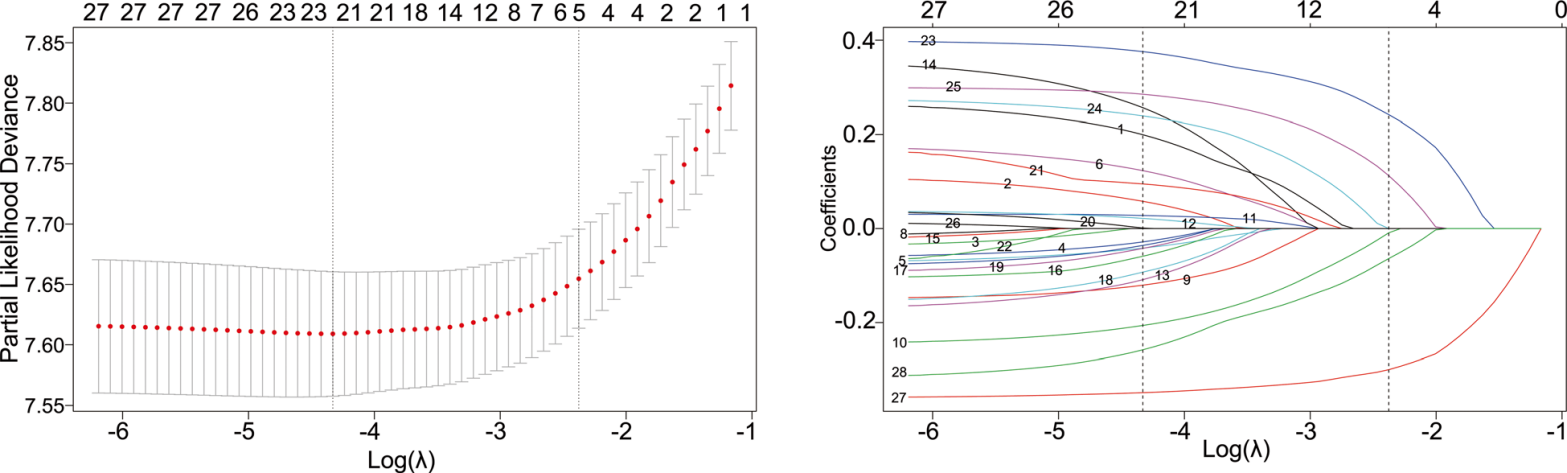
Identification of predictors using the Lasso Cox regression. **a** Selection of tuning parameter (λ) in the LASSO regression using 10-fold cross-validation. The vertical line was plotted using the minimum criteria and the one standard error of the minimum criteria. **b** LASSO coefficient profiles of variables. Each colored line represents a variable in the model. With increases in λ, the coefficient of each variable decreased. At the optimal values log (λ) = − 2.375, five variables (KPS, abdominal distention, edema, QOL, and duration of pain) with a nonzero coefficient were selected

**Fig. 3:**
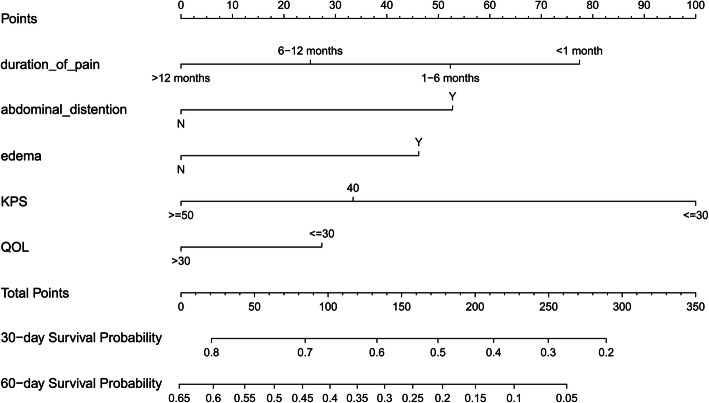
Nomogram predicting the 30-day and 60-day survival probability for patients with gastrointestinal cancer in home palliative care

The optimal tuning parameter λ for LASSO regression with 10-fold cross-validation was 0.093, with log(λ) = − 2.375, following the one standard error of the minimum criteria (Fig. 2a). At the optimal values log (λ), five variables (KPS, abdominal distention, edema, QOL, and duration of pain) with a nonzero coefficient were selected in the LASSO analysis (Fig. 2b). Then the five retained variables were used to construct the nomogram by using multivariate Cox regression. As shown in Table 2, KPS, abdominal distention, edema, QOL, and duration of pain were a panel of significant predictors of overall survival (OS) in patients with gastrointestinal cancer. In the nomogram (Fig. 3), each prognostic variable corresponded to a specific point by drawing a straight line upward to the points axis. After calculating all variables’ points, the total points on the bottom scales that correspond to the 30-day and 60-day survival probability were showed respectively.

**Table 2:** The result of Lasso Cox regression

Variable	β	HR	95%CI	Waldχ2	P
Duration of pain					
1–6 months	−0.185	0.831	0.716–0.965	−2.423	0.015
6–12 months	−0.385	0.681	0.534–0.867	−3.111	0.002
> 12 months	−0.570	0.566	0.357–0.897	−2.422	0.015
Abdominal distention					
Y	0.388	1.475	1.291–1.685	5.714	1.11e-08
Edema					
Y	0.340	1.405	1.205–1.638	4.336	1.45e-05
KPS					
40	−0.490	0.613	0.530–0.708	−6.618	3.64e-11
≥ 50	−0.736	0.479	0.398–0.576	−7.815	5.50e-15
QOL					
>30	−0.201	0.818	0.711–0.940	−2.834	0.005

**Fig. 4:**
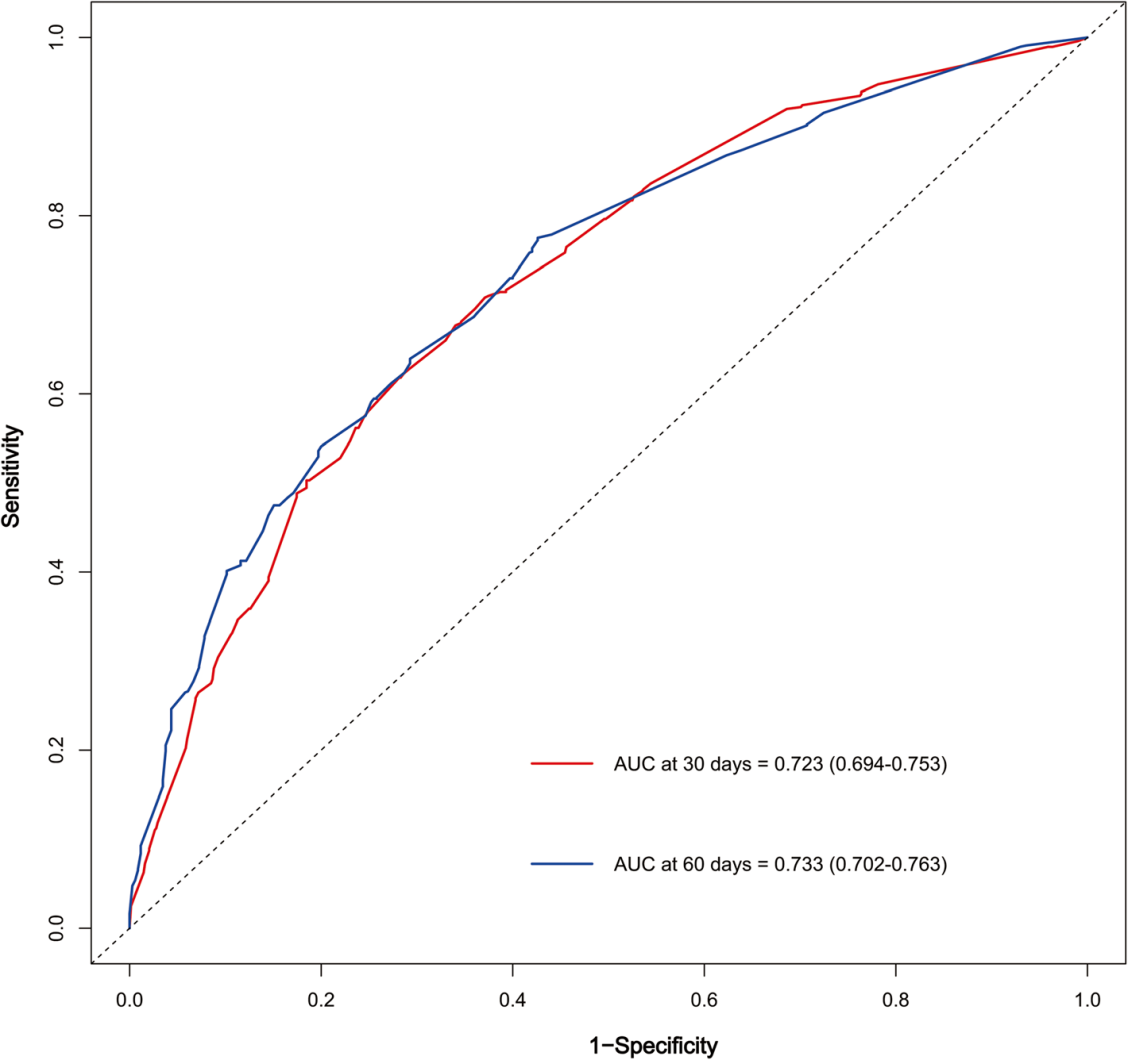
Receiver operating characteristic (ROC) curve analysis for the nomogram in the training dataset

**Fig. 5:**
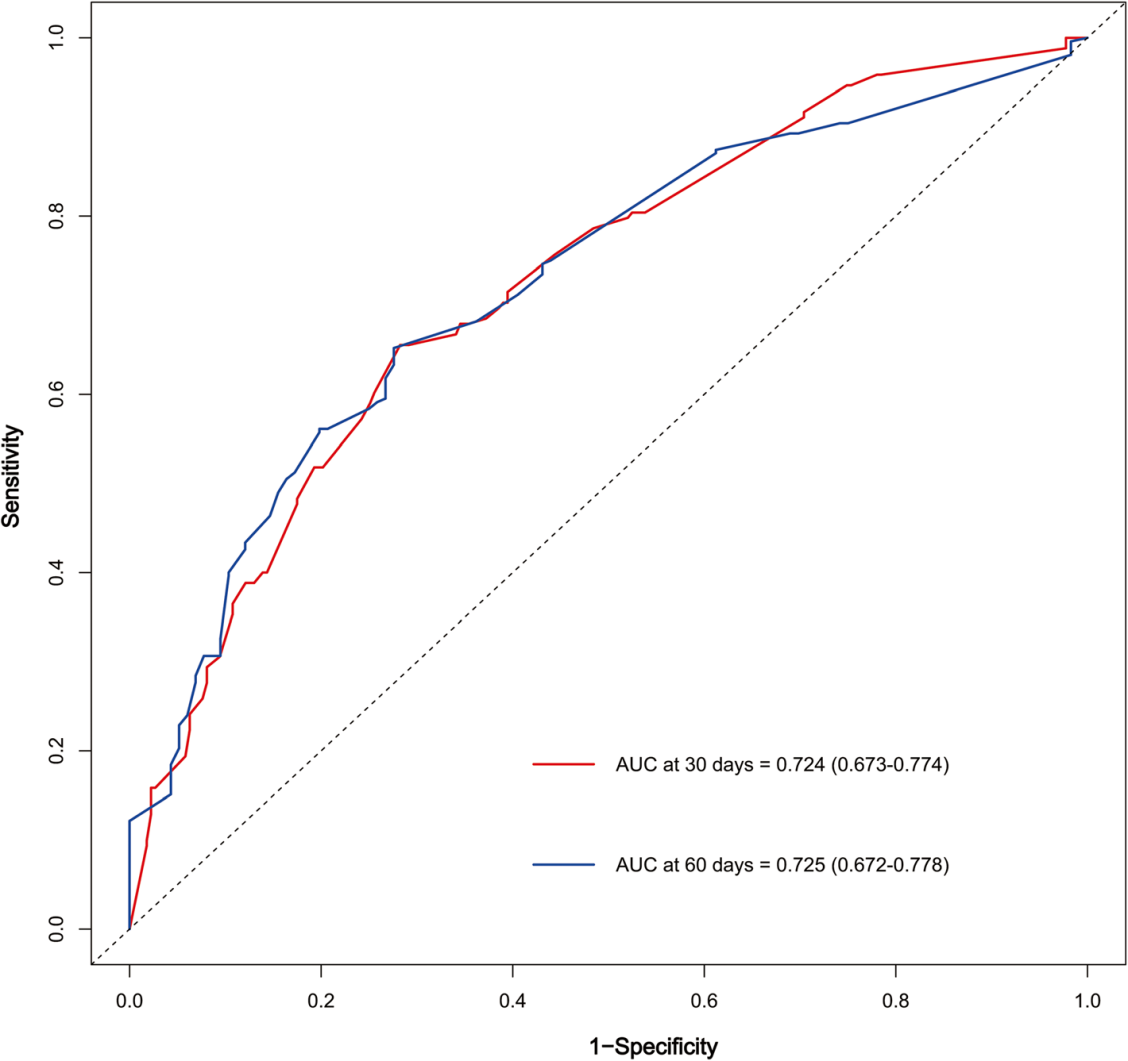
Receiver operating characteristic (ROC) curve analysis for the nomogram in the testing dataset

**Fig. 6:**
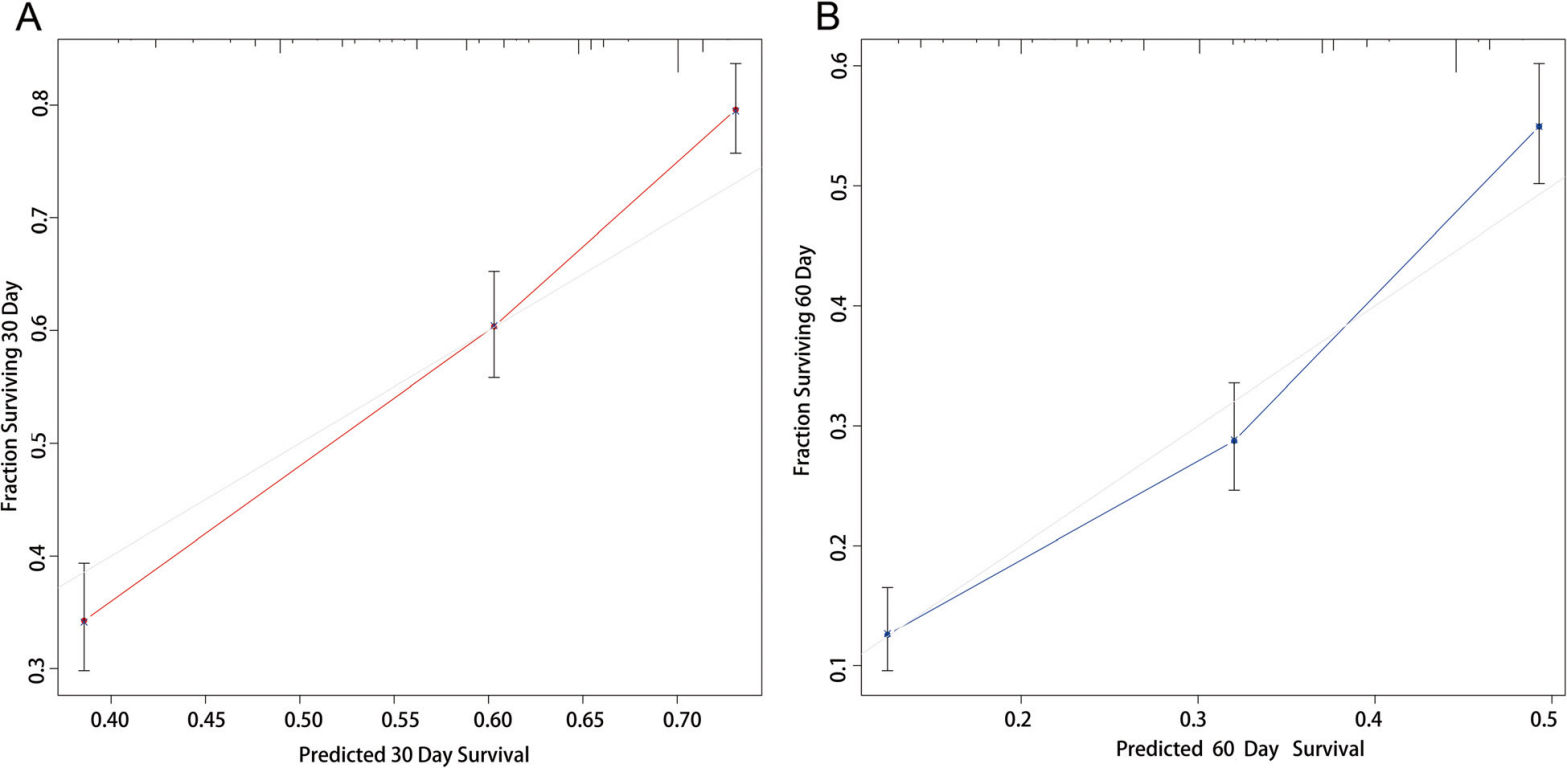
Calibration curves for predicting (**a**) 30-day and (**b**) 60-day overall survival in the training dataset

**Fig. 7:**
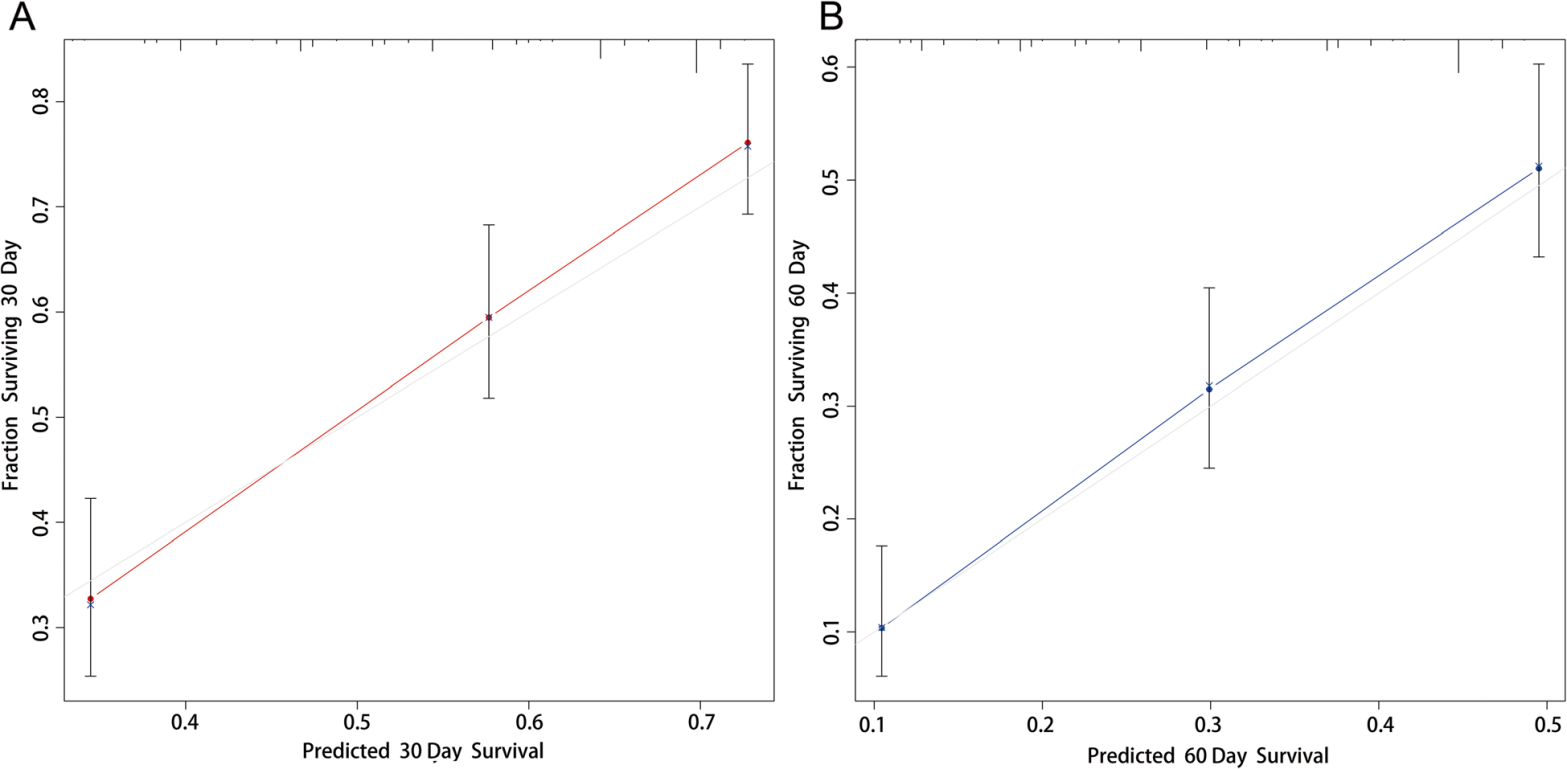
Calibration curves for predicting (**a**) 30-day and (**b**) 60-day overall survival in the testing dataset

We examined the performance of our predictive nomogram by employing both discrimination and calibration assessments. The receiver operating characteristic (ROC) curve analysis showed quite useful discrimination in both the training and testing dataset. As shown in Fig. 4, the AUC value was 0.723 (95% confidence interval, 0.694–0.753) at 30 days and 0.733 (95% confidence interval, 0.702–0.763) at 60 days in the training dataset. Similarly, the AUC value was 0.724 (0.673–0.774) at 30 days and 0.725 (0.672–0.778) at 60 days in the testing dataset (Fig. 5). To assess the calibration of the prognostic nomogram, we compared the predicted 30-day and 60-day survival probabilities to the actual 30-day and 60-day survival probabilities. As shown in Fig. 6 and Fig. 7, the calibration curves revealed good agreement between the predicted and observed probabilities.

## Discussion

Previous literature regarding home hospice care in China does not differentiate between specific cancer groups [23, 24]. However, several studies have shown that prognosis information varies by cancer types [21, 36, 37]. As a developing country, because of environmental pollution and the lack of early diagnosis and treatment, high incidence occurs in gastrointestinal cancer with poor prognosis in China [1]. This is the first time to analyze gastrointestinal cancer-specific prognostic factors that influence patients’ survival and build a model to accurately predict survival time in home hospice care.

Compared with previous studies, the application of LASSO Cox regression with cross-validation enabled us to develop a more parsimonious predictive model with superior performance. For example, Zhou LJ et al. identified 10 prognostic variables to develop a simple Chinese Prognostic Scale (ChPS) with 65.4% accuracy in the testing set by using traditional multivariable regression [24]. In our study, we used the LASSO analysis to identify 5 preditors from all 28 initial variables. And the evaluation of our predictive model showed quite useful discrimination and good agreement calibration in both the training and testing dataset. This is consistent with the opinion that the LASSO have a better performance against the traditional multivariable regression since it can perform both variable selection and penalization [38]. Penalized regression is utilized to avoid model overfitting by using a loss function or penalty term that is added to the objective function to control the complexity of the model [31]. In clinical scenarios, a more selective model would be preferred because it could save time and resources, by avoiding collection of less useful data. Besides, our predictive nomogram has the advantage of not utilizing laboratory measures, which are difficult to obtain in hospice patients.

Many studies suggested that performance status along with some clinical symptoms could improve the prediction of survival for terminal cancer patients [24, 39, 40]. This parallels our findings. In our prognostic nomogram, there are five predictors including KPS, abdominal distention, edema, QOL, and duration of pain. Among these, KPS, the recognized tool to evaluate performance status, has been found to be reasonably reliable in survival prediction for patients with advanced cancer even when scores were as low as 50 [40, 41]. Poor performance status is associated with short survival. As shown in our nomogram, the lower KPS patients were scored, the higher points they receive, indicating the worse their 30-day and 60-day overall survival. Similarly, a patient obtained a QOL score 30 or lower has worse probability of survival than those were scored higher than 30. For the predictor that “duration of pain” in our nomogram, the shorter duration, the higher points, the worse probability of survival. This is likely because those patients who experience acute pain usually have sever disease progression, which leads to shorter survival. Furthermore, we should note that the symptoms (edema, abdominal distention and duration of pain) included in our nomogram were not exactly the same as those included in previous studies [42]. This may be due to the different characteristics of the samples included in studies. As Glare reported that there appeared to be apparent differences in prognostic factors between those predicted survival less than 3 months vs. those predicted survival ranging in the 3–12 months [43]. In addition, KPS and QOL scores are generally low in our study. That’s possibly because the performance and QOL status of patients with advanced cancer in home hospice care is generally poor.

This study is certainly limited because it was performed using a retrospective database from one hospice center in China. First, the AUC is quite acceptable, but not outstanding. It may be partly due to the retrospective nature of this study and the lack of our ability to capture all useful predictors and the precision of each predictor. The data such as symptoms were collected as “present” or “absent” from the case records. Second, the optimal method of validation for our predictive model is to use a separate dataset from another center. A potential solution is to prospectively perform a multicenter study, though this is time-consuming and potentially unfeasible. Moreover, patients with any missing data were excluded from our study analysis, which may affect the robustness of the model to some extent. Last but not least, we only included research objects who died within six months, which seems to cause subject bias. However, the clinical reality also needs to be considered. Precious few patients survived more than six months in the hospice unit, and they were mostly in the early stages of cancer. Those patients chose hospice treatment mainly because of financial difficulties.

## Conclusion

To our knowledge, this is the first application of LASSO Cox regression with cross-validation to produce a gastrointestinal cancer-specific nomogram to predict the probability of survival at 30 days and 60 days in home hospice care patients in China. Our nomogram may be a useful non-lab clinical tool that needs prospective multicenter validation as well as testing with Chinese clinicians in charge of hospice patients with gastrointestinal cancers to assess acceptability and usability.

## Supplementary Information

**Additional file 1: Supplementary Table 1**. The distribution of GI cancers for the training and testing dataset.

## Data Availability

The datasets used and analysed during the current study are available from the corresponding author on reasonable request.

## Abbreviations

LASSO: Least absolute shrinkage and selection operator
AUC: Area under receiver operating characteristic curve
ROC: Receiver operating characteristic
IQR: Interquartile ranges
OS: Overall survival
KPS: Karnofsky Performance Scale
QOL: Quality-of-life
NRS: Numeric rating scale
PaP: Palliative Prognostic
D-PaP: Delirium-PaP
PPS: Palliative Performance Scale
PPI: Palliative Prognostic Index
mGPS: Modified Glasgow Prognostic Score
PiPS: Prognosis in Palliative Care Study
ChPS: Chinese Prognostic Scale
