# Supplementary material for: A non-lab nomogram of survival prediction in home hospice care patients with gastrointestinal cancer
Source: BMC Palliat Care. 2020 Dec 7;19:185. doi: 10.1186/s12904-020-00690-2 (PMC7722330; doi:10.1186/s12904-020-00690-2)
Supplement: Supplementary file 1 — Additional file 1: Supplementary Table 1. The distribution of GI cancers for the training and testing dataset. [file 12904_2020_690_MOESM1_ESM.docx]

Supplementary Table 1. The distribution of GI cancers for the training and testing dataset

| Diagnosis | Overall  (n, %) | Training set  (n, %) | Testing set  (n, %) | P= 0.405 |
| --- | --- | --- | --- | --- |
| Esophageal cancer | 402 (24.8) | 304 (25.0) | 98 (24.3) |  |
| Gastric cancer | 240 (14.8) | 174 (14.3) | 66 (16.3) |  |
| Colorectal cancer | 385 (23.8) | 296 (24.4) | 89 (22.0) |  |
| Liver cancer | 410 (25.3) | 311 (25.6) | 99 (24.5) |  |
| Gallbladder cancer | 44 (2.7) | 27 (2.2) | 17 (4.2) |  |
| Pancreatic cancer | 98 (6.1) | 73 (6.0) | 25 (6.2) |  |
| Others* | 39 (2.4) | 29 (2.4) | 10 (2.5) |  |

Others*: Cancers in other parts of the digestive system, such as the small intestine.
